# Evidence for the Induction of Key Components of the NOTCH Signaling Pathway via Deltamethrin and Azamethiphos Treatment in the Sea Louse *Caligus rogercresseyi*

**DOI:** 10.3390/ijms17050304

**Published:** 2016-05-12

**Authors:** Sebastian Boltaña, Jaqueline Chávez-Mardones, Valentina Valenzuela-Muñoz, Cristian Gallardo-Escárate

**Affiliations:** Laboratory of Biotechnology and Aquatic Genomics, Interdisciplinary Center for Aquaculture Research (INCAR), Department of Oceanography, University of Concepción, Concepción 4030000, Chile; sboltana@udec.cl (S.B.); jchavez@udec.cl (J.C.-M.); valevalenzuela@gmail.com (V.V.-M.)

**Keywords:** *Caligus rogercresseyi*, deltamethrin, azamethiphos, RNA-seq, NOTCH signaling pathway, ABC transporters

## Abstract

The extensive use of organophosphates and pyrethroids in the aquaculture industry has negatively impacted parasite sensitivity to the delousing effects of these antiparasitics, especially among sea lice species. The NOTCH signaling pathway is a positive regulator of ABC transporter subfamily C expression and plays a key role in the generation and modulation of pesticide resistance. However, little is known about the molecular mechanisms behind pesticide resistance, partly due to the lack of genomic and molecular information on the processes involved in the resistance mechanism of sea lice. Next-generation sequencing technologies provide an opportunity for rapid and cost-effective generation of genome-scale data. The present study, through RNA-seq analysis, determined that the sea louse *Caligus rogercresseyi* (*C. rogercresseyi*) specifically responds to the delousing drugs azamethiphos and deltamethrin at the transcriptomic level by differentially activating mRNA of the NOTCH signaling pathway and of ABC genes. These results suggest that frequent antiparasitic application may increase the activity of inhibitory mRNA components, thereby promoting inhibitory NOTCH output and conditions for increased resistance to delousing drugs. Moreover, data analysis underscored that key functions of NOTCH/ABC components were regulated during distinct phases of the drug response, thus indicating resistance modifications in *C. rogercresseyi* resulting from the frequent use of organophosphates and pyrethroids.

## 1. Introduction

*Caligus rogercresseyi*, like *Lepeophtheirus salmonis*, is a marine ectoparasite that infests farmed and wild salmonids, causing high economic losses for the industry [1,2]. The current control method for caligidosis, or infection with *C. rogercresseyi*, is the use of chemical treatments. Insecticides commonly used in salmonid farming include avermectins (e.g., emamectin benzoate), pyrethroids (e.g., deltamethrin (DM)), organophosphates (e.g., azamethiphos (AZA)), and hydrogen peroxide [3]. Apart from the environmental damage inherent to any large-scale therapeutic drug administration, pesticide resistance is a growing concern. For example, different delousing drug also induce treatment resistance in sea lice [3,4,5,6].

The NOTCH signaling pathway is a positive regulator of ATP-binding cassettes (ABC) transporter subfamily C (ABC C) expression. In arthropods, ATP-driven efflux transport systems have been linked with resistance to pesticides and other drugs [7,8,9,10]. However, there are few studies on the NOTCH and ABC interactome in marine ectoparasites treated with delousing drugs, and insight on this subject could provide important information regarding the generation and modulation of pesticide resistance. NOTCH is a heterodimeric Type 1 transmembrane receptor coded by one of four NOTCH genes (*Noctch1*–*NOTCH4*) [11]. Interaction with these receptors activates γ-secretase and the A Disintegrin and Metalloprotease complexes, resulting in the cleavage of the intracellular C-terminal fragment of NOTCH (*N^IC^*). Following cleavage, N^IC^ is translocated to the interior of the nucleus, where it changes the *CBF1/RBP-Jk*/Suppressor of Hairless/LAG-1 (*CSL*) transcription factor from a transcriptional repressor to a transcriptional activator through the substitution of a repressor complex with an activator complex [12]. The *CSL/N^IC^* activation complex trans-activates various target genes, including genes from *HES/HEY* family members that actively repress a tissue-specific NOTCH-signaling pathway. Modifications in the expressional regulation of the NOTCH protein, ligands, and targets have been described in a number of tumor tissues in humans [13]. Intracellular forms of NOTCH have also been implicated in the resistance of thymocytes to glucocorticoids [14]. Moreover, N^IC^ participation has been reported in resistance to adriamycin, cisplatin, etoposide, and taxol in MCF7 cells, MOLT4 cells, breast adenocarcinoma cell lines, and infantile lymphoma T-cells, respectively [15].

The ABC transporters are membrane proteins that mediate the unidirectional transport of biological substrates linked to ATP hydrolysis. These transporters are recognized members of the multidrug resistance protein family [16]. The superfamily of ABC transporters in invertebrates is divided into eight subfamilies (*ABC A*–*ABC H*), which are differentiated by domain and sequence architectures [17,18]. Of note, the B, C, and G subfamilies have direct roles in processes of detoxification [19]. In humans, the *ABC C1* proteins transport a wide range of xenobiotics, physiological substrates, and other components [20]. The overexpression of *ABC C1* in tumor tissues has been clearly associated with clinical drug resistance in cases of intestinal and esophageal cancers [13]. In terms of pesticide resistance, there is a close relationship in *L. salmonis* between the expression levels of P-glycoprotein, a member of the *ABC B* subfamily, and emamectin benzoate resistance [21]. Moreover, expressional regulation of the *ABC C1* gene is associated with the promoter of the 5′UTR through various transcription factors, including the GC box, activator protein 1, and E-box elements [22]. Furthermore, the promoter region of *ABC C1* interacts with the transcription factor core-binding factor 1, an interaction activated by *N^IC^* in association with the NOTCH signaling pathway [23].

The extensive use of organophosphates and pyrethroids in the aquaculture industry has negatively impacted parasite sensitivity to the delousing effects of antiparasitics, especially in sea lice such as *L. salmonis* [6,24]. However, little is known about the molecular mechanisms underlying drug transport that are affected by these chemicals. This situation is partly due to a lack of genomic and molecular information, especially in *C. rogercresseyi*. Next-generation sequencing technologies have revolutionized the fields of genomics and transcriptomics, providing an opportunity for rapidly and cost-effectively generating genome-scale data [25]. Considering the negative impacts of caligidosis and the increasing instance of resistance, it is highly relevant to study the transcriptomic performance of *C. rogercressey* treated with DM and AZA to establish the effects of drug concentrations on mRNA abundances in key molecular processes, such as in the NOTCH signaling pathway and ABC transporters. RNA-seq analysis determined that the sea louse *C. rogercresseyi* specifically responded to both delousing drugs at a transcriptomic level by differentially activating mRNA of the NOTCH signaling pathway and of ABC genes. Groucho and HDAC are key regulators of NOTCH signaling pathway mediating the transcriptional repression of NOTCH downstream, the present study shows for the first time its mRNA regulation and interaction with NOTCH gene cassettes after drug treatment. Additionally, the expression of single nucleotide polymorphisms (SNPs) was predicted for NOTCH and ABC components to facilitate gene mapping and genetic variation analysis in *C. rogercresseyi* exposed to DM and AZA.

## 2. Results

### 2.1. Identification and Expression of NOTCH Signaling Pathway Gene Components from C. rogercresseyi

**Figure 1:**
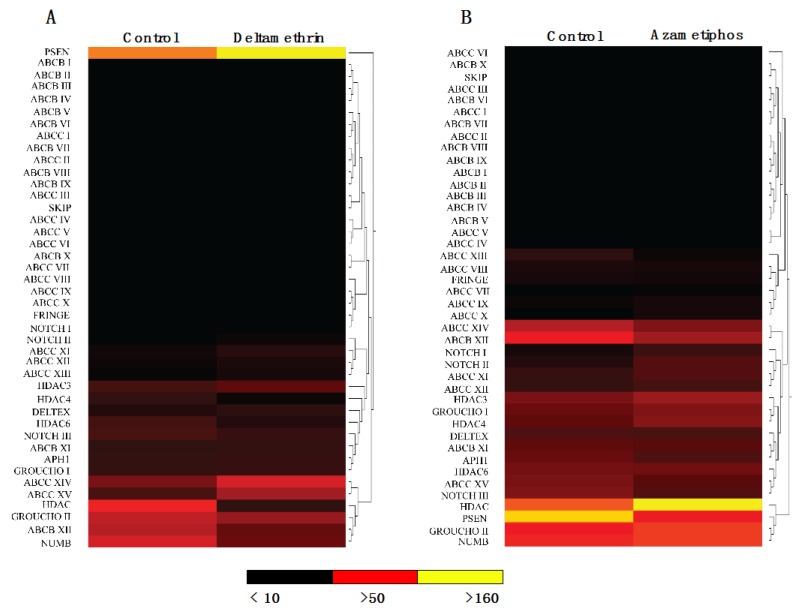
Heat map analysis of NOTCH signaling pathway components. Heat map generated by RNA-seq analysis for components of the NOTCH signaling pathway in adult *C. rogercresseyi* treated with antiparasitics. (**A**) Deltamethrin and (**B**) Azamethiphos. Hierarchical clustering was performed using the Euclidean distance matrix and complete linkage method, while the Manhattan metric distance was used for hierarchical cluster analysis. Analyses were conducted in Cluster3/TreeView open software.

From the *de novo* assembly of transcripts obtained from sequencing six developmental stages of *C. rogercresseyi* [25,26] 15 transcripts participating in the distinct stages of NOTCH pathway signaling were identified, while 27 transcripts were annotated for ABC transporters (Table 1). This information allowed establishing a putative NOTCH signaling pathway in *C. rogercresseyi*. The identified transcripts had *E*-values between 1 × 10^−29^ and 0, with high homology to sequences submitted in the NCBI gene bank for arthropods, such as *Daphnia pulex* (ID: 6669), *Drosophila melanogaster* (ID: 7227), and *L. salmonis* (ID: 72036). *In silico* analysis of NOTCH signaling pathway expression showed significant expressional changes in pathway components in individuals exposed to the delousing drugs. Histone deacetylase (*HDAC*) was upregulated in the group challenged by DM, while *NOTCH1, 2* were slightly upregulated in this same group (Figure 1A). The upregulated components of the NOTCH signaling pathway modulated by AZA included *NOTCH1, 2* (Figure 1B). There was high presenilin-1 (*PSEN1*) expression in the control group, but this significantly decreased in the group exposed to AZA. *PSEN* also was upregulated in the control and decreased after the DM treatment, suggesting that the both drugs AZA and DM promote the decrease in its mRNA abundance. RNA-seq analysis showed that components of the NOTCH signaling pathway modulated by AZA presented differences in expression compared to the control group. The *GROUCHO ii*, *HDAC4*, *HDAC6*, *NOTCH (1–2)*, and *NUMB* genes were differentially expressed in the AZA group compared to the control. The percentage of sea lice that died during treatment is indicated in Table 2. The survival rate of *C. rogercresseyi* was lower for 3 ppb of DM than for 3 ppb of AZA. Large differences in survival were also found between sea louse sexes after DM treatment, with 3 ppb inducing the death of all male sea lice.

**Figure 2:**
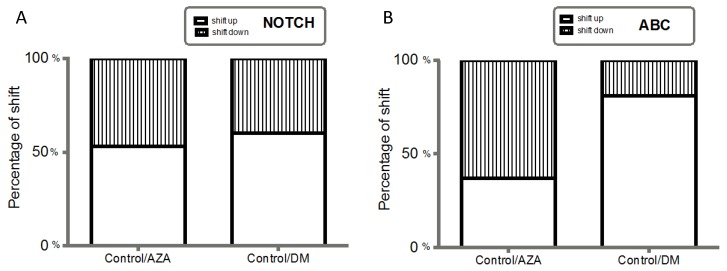
Regulation of NOTCH/ABC components. (**A**) Percentile directional shift (Control *vs.* Deltamethrin (DM)); (**B**) Percentile directional shift (Control *vs.* Azamethiphos (AZA)). Hatched and white bars represent down and upregulated transcripts, respectively, and percentile increases represent mean mRNA transcript abundance (% increase or decrease, respectively) of representative regulatory pathways.

To determine if the delousing drugs simply shifted the global gene expression profile of the NOTCH and ABC pathways by advancing or delaying the response, the maximum abundance levels of mRNA transcripts were compared over a period post-delousing challenge. Transcript analyses highlighted significant, dose-dependent differences in specific mRNAs (Figure 2A,B). Notably, significant interactions were identified between mRNA pathways when evaluated after AZA and DM exposure. The observed differences in mRNA abundance were not a consequence of a temporal shift, but rather reflected specific drug-dependent increases in transcript abundances of the ABC genes and NOTCH pathway (Figure 2A). This suggests increased transcription focus and functional specialization, as well as a strong effect of delousing drugs on these processes. In turn, this increased response indicates that AZA and DM drive modifications in the regulation of downstream NOTCH components, as well as in the mRNA abundances of ABC genes, suggesting that NOTCH/ABC regulation plays a role in drug resistance in Caligus species. Furthermore, the directional shift of transcripts regulated within NOTCH/ABC mRNAs was measured. The addition of DM as a variable showed that 70% of the transcripts within ABC clusters were directionally increased as compared to controls, and these transcripts were linked to increased abundance variation, signifying a change in functional NOTCH/ABC output (Figure 2B).

### 2.2. Differentially Expressed Genes of the NOTCH Signaling Pathway from C. rogercresseyi

**Figure 3:**
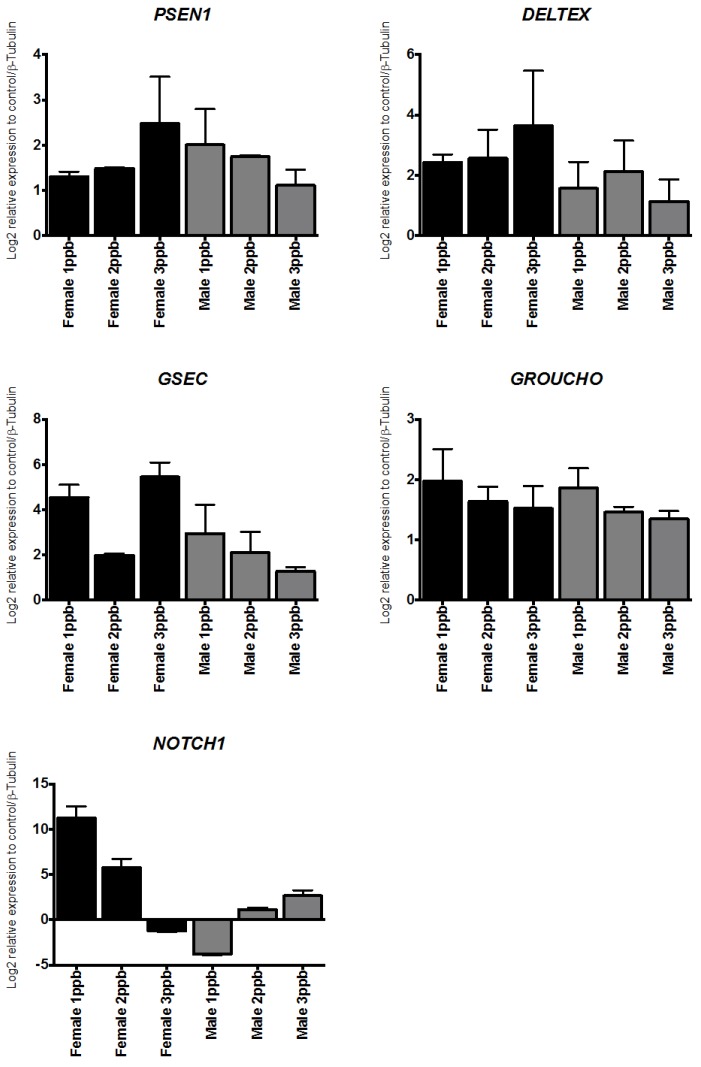
Transcriptional response of NOTCH signaling pathway components. Transcriptional response of NOTCH signaling pathway components in *C. rogercresseyi* exposed to deltamethrin. Each bar represents mean (±SD) expression levels normalized to *β*-tubulin (*n* = 9).

**Figure 4:**
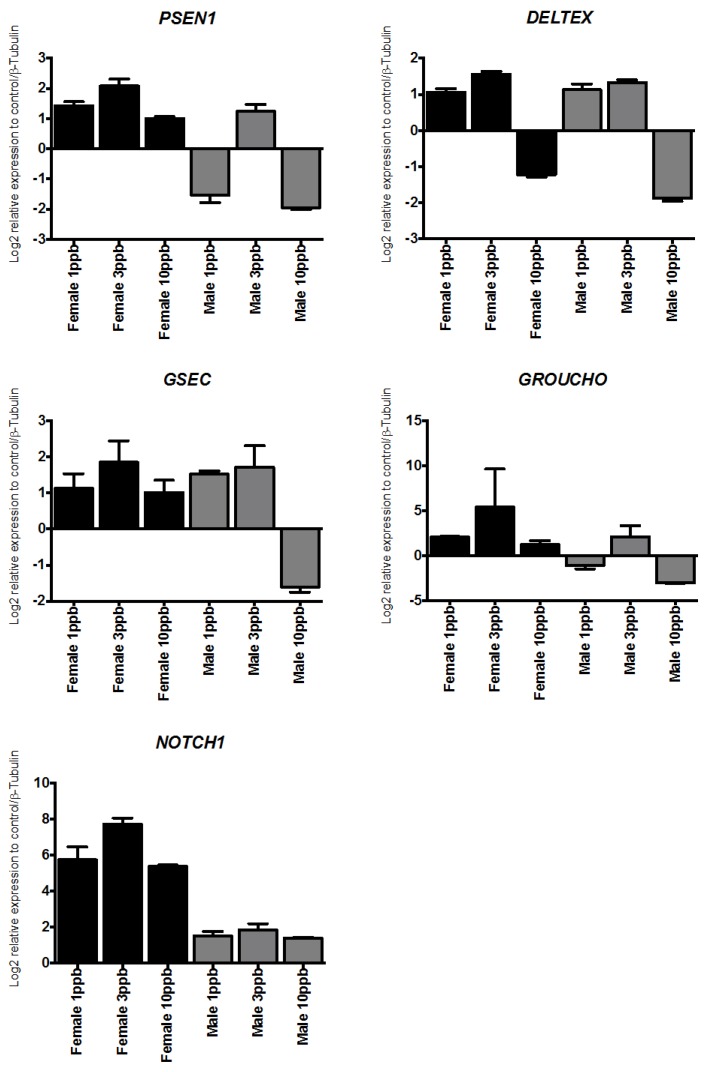
Transcriptional response of NOTCH signaling pathway components. Transcriptional response of NOTCH signaling pathway components in *C. rogercresseyi* exposed to azamethiphos. Each bar represents mean (±SD) expression levels normalized to *β*-tubulin (*n* = 9).

Real time-qPCR was used to examine whether DM or AZA were able to trigger differences in the abundances of ABC mRNA related to the downstream NOTCH pathway or ABC transporters. The obtained results showed that both delousing drugs triggered an overall increase of gene expression and that these genes were differentially regulated in a dose-dependent manner (e.g., *PSEN1* and *GSEC*). As shown in Figure 3 and Figure 4, AZA only induced the expression of downstream NOTCH components depending on the sex. However, both treatments induced a weak response at 10 ppb in males and females. DM induced a large increase of *PSEN11*, *DELTEX*, *PSEN1EN*, and *GROUCHO*, with maximum expression at 3 ppb in males and between 1–2 ppb for females, with expression gradually decreasing in females until 3 ppb. AZA increased the expression of NOTCH pathway components, with maximum expression at a concentration of 3 ppb, decreased expression at 1 ppb, and nearly undetectable expression at 10 ppb (Figure 4).

**Figure 5:**
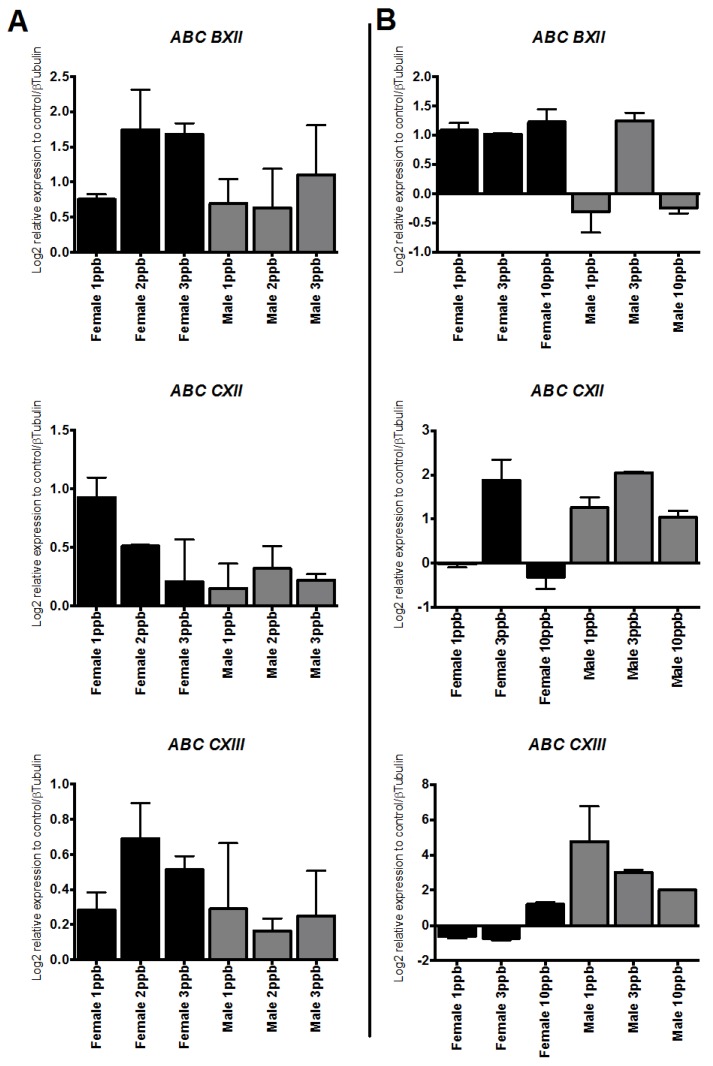
Transcriptional response of ABC membrane transporters in *C. rogercresseyi* exposed to two antiparasitics. (**A**) Deltamethrin; and (**B**) Azamethiphos. Each bar represents mean (±SD) expression levels normalized to β-tubulin (*n* = 9).

Using RT-qPCR, both AZA and DM (1–3 or 10 ppb) promoted mRNA increases of the *ABC* genes *CXII*, *CIII*, and *BXII*. Additionally, ABC transporters seemed to be highly influenced by AZA, with *ABC CXII, CIII* and *BXII* gene expressions closely correlated to sex, mostly increased in males, but not to dose (Figure 5). In the DM group, the expression of *ABC* transporters had a response opposite to that observed with AZA treatment. DM increased the mRNA levels of *ABC* genes *CXII*, CXIII, and BXII with the three doses and for both sexes, in contrast to that observed with AZA. AZA triggered increased mRNA abundance, mainly in males, for *ABC BXII* and *CXIII*, with females displaying maximum expression at 1–2 ppb, with decreasing expression until 3 ppb (Figure 5).

### 2.3. Principal Component Analysis (PCA)

The global expression profiles of *C. rogercresseyi* exposed to the delousing drugs DM and AZA were compared. Transcripts were scored positively for reliable annotation among all samples. These transcripts were used for statistical analysis to find mRNAs with abundance levels that significantly differed between the two treatments. DeDaL was used to create a DDL layout for a group of *C. rogercresseyi* genes involved in the downstream NOTCH pathway. Hierarchal cluster analysis and PCA were applied to determine the contribution that each gene had in the response to treatment with DM and AZA.

**Figure 6:**
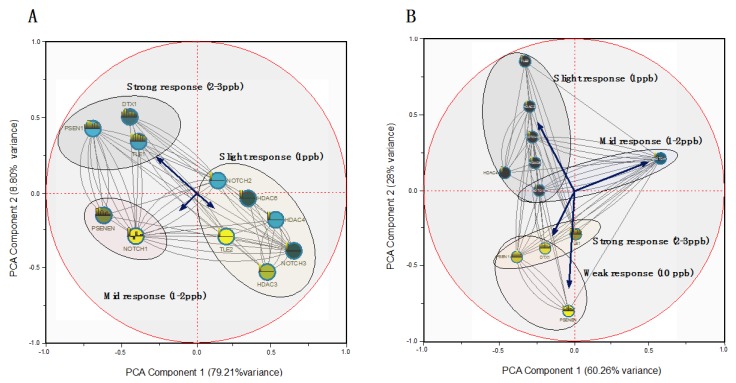
Interaction network between NOTCH/ABC genes involved in delousing drug treatments. Different node colors indicate distinct mRNA regulations during chemical exposure. Principal component analysis (PCA) was performed on the network-smoothed profile through DDL computation using an elastic map (elmap) algorithm for calculating the non-linear principal manifold. A factorial map of PCA was constructed for data from all commonly expressed transcripts in each pathway, represented as a point for each delousing drug, (**A**) Deltamethrin and (**B**) Azamethiphos. The coordinates of the arrow on the ordination axes represent the values of Pearson’s correlation coefficient between the gene expression and the drug concentration used in each treatment. The percentage of variance explained by each principal component is indicated in parentheses.

The DeDaL-PCA Cytoscape plugin combined the classical and advanced data dimension reduction methods with the algorithms of network layout inside the Cytoscape environment. The genetic interactions between the analyzed genes using RT-qPCR and the corresponding epistatic profiles were selected from the global expression profile provided by RNA-seq. Definitions of the NOTCH pathway were taken from the KEGG database [27]. Figure 6A,B shows the differences between the standard organic and PCA-based DDL layouts for this small network of genetic interactions. These layouts were computed without double-centering the matrix data to take into account tendencies of genes to interact with a smaller or larger number of other genes, thereby estimating the effects that AZA and DM concentrations had on the mRNA abundance of NOTCH pathway components.

Both PCAs (Figure 6A,B) denoted the differences between delousing drugs, where the concentrations used in each treatment could easily be separated, in addition to identifying a threshold concentration used for the treatments. First, the local NOTCH genes *TLE2, HDAC3*, *NOTCH2*, and *NOTCH1* had distinct positions in each PCA layout. Second, PCA-based DDL roughly grouped the genes according to involvement in each drug concentration. The PCA revealed two main specific global scale patterns based on the different responses of *C. rogercresseyi* when treated with DM or AZA (Table 2). The correlated variability pattern of the expressed genes was prominently different between DM and AZA. In addition to the global separation that defined the responses to the two drugs, an inverse sex-dependent pattern was found when comparing the different associated genes (Figure 6A,B). For example, AZA concentrations had a strong effect on the NOTCH pathway components (Figure 6B). Moreover, some genes, such as *PSEN1EN, PSEN11*, and *DELTEX*, showed a weak expression pattern at a concentration of 10 ppb (*i.e.*, negative threshold of activation). This was in contrast to the strong response triggered at concentrations of 1–3 ppb. In both treatments, the two principal factors explained close to 90% of expression variability (Figure 6A,B), and a similar dose-dependent effect of each treatment on the expression profile of the downstream NOTCH pathway was suggested.

### 2.4. Single Nucleotide Polymorphisms (SNPs) Mining

A total of 54 SNPs were identified in NOTCH signaling components in *C. rogercresseyi* for both sexes (Table 3). In NOTCH components, the identified variations were synonymous or present in the 3′ or 5′UTR (Table 3). Just three contigs presented SNPs in the open reading frame and 3′UTR. For contigs annotated for *DELTEX*, 11 SNPs were related with synonymous variations while three were non-synonymous. Four SNPs were present in the open reading frame and 3′UTR. Despite these results, more studies are necessary to determine the function of non-synonymous variations in NOTCH components, as well as the possible association of these with delousing drug resistance.

## 3. Discussion

Several studies have identified DM and AZA as effective pesticides in the control of caligidosis, triggering, among other processes, the generation of reactive oxygen species [28]. However, little information is available about the effects of these pesticides on the transcriptomic response of the parasite and the correlation of this response with drug resistance. This study is the first to characterize the NOTCH signaling pathway and ABC transporters in *C. rogercresseyi* and the regulation of these under treatment with pesticides.

Different delousing drugs promote changes in the transcriptional profile in the sea lice *L. salmonis* and *C. rogercresseyi* [4,27,29,30]. By systematically dissecting the impact and combination of treatment parameters (sex and dose), the present data revealed a significant remodeling of the *C. rogercresseyi* transcriptome, thus emphasizing the strong effects of delousing drugs. In *C. rogercresseyi*, transcriptomic profile modifications occur in response to developmental stages, reproductive phases, or the maintenance of steady state transcriptional activity [26,28,29,30]. In sea lice, exposure to delousing drugs such as DM or AZA results in a shift from a steady state to a functional reactive state (*i.e.*, production of reactive oxygen species) through the secretion of peroxinectin [28,29,30].

The present study demonstrated a strong response to delousing chemicals by the entire transcriptome, as well as by key molecular process closely linked with pesticide resistance, such as in the NOTCH/ABC pathways. RNA-seq analysis identified a dose-dependent, differential regulation of mRNAs following exposure to the delousing drugs AZA and DM. A similar phenomenon was observed for response magnitude; however, in most cases, DM triggered higher expressions of NOTCH pathway components compared to AZA exposure. Interestingly, 10 µg/mL was the threshold AZA concentration needed to activate downstream NOTCH components. These data support previous research on delousing chemicals, which found similar activation thresholds [28]. Other studies have closely linked the transcriptomic response of *L. salmonis* and *C. rogercresseyi* with the generation of resistance to emamectin benzoate, showing that P-glycoprotein gene expression slightly increases after 24 h of exposure to 10 ppb of this avermectin [21,31]. Together, these results suggest that although there are differences in drug compositions, a common reaction threshold to delousing drugs exists.

Considering this, the present analyses denote the importance of concentration and sex on response intensity (fold-change) at a molecular level. Varied sensitivity is likely conferred by the participation of different ABC genes and NOTCH receptors in the drug-Caligus interaction, and/or the accumulative signaling intensity (*i.e.*, threshold) of the group of receptors involved in the reaction mechanism. Integrative analysis of transcriptome data through computational and analytical approaches further supports this observation by extending the interpretative value of a one-dimensional gene list into regulatory modules and interaction networks [32]. Comparison of drug-reaction transcriptomes at different levels, including of pathway-concentration interactions and PCA-module analyses, demonstrated significantly different modifications in the magnitude and intensity of measured responses to the delousing drugs.

Transcriptional analysis showed that AZA and DM were able to trigger a minor increase in the mRNA abundances of *PSEN1* and *APH-1*. *Presenilin-1* is a catalytic subunit of the γ-secretase complex and has been principally described as a modifiable component of the NOTCH signaling pathway [33]. The slight changes in the mRNA abundances of both subunits suggest that NOTCH cleavage cannot take place. Moreover, the antiparasitics seemed to affect the negative regulator of the NOTCH receptor by triggering changes (downregulation) the mRNA abundance of *NUMB* (see Figure 1 and Table 1). This protein has a phosphotyrosine-binding domain in the N-terminal and is linked by this domain to the cell membrane location that contributes to the role of *NUMB* in the control of intracellular NOTCH trafficking [21,34].

Additionally, RNA-seq analysis identified two proteins of the NOTCH repressor complex, *HDAC* and *GROUCHO*. *GROUCHO* is a protein with five domains, two of which are highly conserved at the N- and C-terminals (Gln and Trp-Asp). These domains have essential functions in interacting with a variety of DNA-binding proteins and in mediating the transcriptional switch-off of the downstream NOTCH pathway [35]. Particularly, *HDAC* is an enzyme with a multiprotein-complex that suppresses the CSL factor of NOTCH receptors [36].

In the present study, *HDAC* showed differential and contrasting responses to each drug; DM promoted a slight increase in *HDAC* mRNA abundance while AZA triggered a high increase in *HDAC* mRNA. These results suggest a repressor role of the CSL transcription factor in the presence of organophosphates. The ABC transporters were also promoted during AZA exposure. These results are in line with a previous report by Cho *et al.* [23], showing that components of the NOTCH signaling pathways are closely associated with the ABC transporters and that both are stimulated during drug exposure [23,37]. Collectively, these data suggest a significant and similar effect of both chemicals, in addition to indicating that frequent organophosphate and pyrethroid use could be promoting parasite resistance through the inhibition of NOTCH/ABC gene components.

Interestingly, generalized transporter-responsive transcriptional changes were strongly evident until the dose of the organophosphate or pyrethroid was increased. As detailed above, the prime candidates in emerging drug resistance are genes involved in the ABC and NOTCH signaling pathways. Indeed, the current results support the hypothesis that treatment drugs have dose-dependent effects on individuals, such as with an upregulation of mRNAs related to inhibitory components of the NOTCH/ABC genes. The interactions within this essential regulation-receptor system could play a key role in sea lice drug resistance [9,38] such as observed for the effect of AZA and DM on cytochrome P450 [10].

Multiple mRNA transporter-related components linked to apoptosis, mitochondrial transport, oxidative stress, and glycolysis are affected by organophosphates and pyrethroids. A significant body of work describes delousing drug-induced cellular toxicity [13,14,15,33,39]. This is in addition to previous studies that have shown organophosphates and pyrethroids capable of causing mitochondrial oxidative stress in various invertebrate species [6,28]. However, little information is available on NOTCH/ABC mRNA regulation and the putative role of this pathway in pesticide resistance. It is possible that treatment-induced mRNA regulation is restricted in terms of the drugs and concentrations used. Therefore, the presented transcriptome signaling for the NOTCH/ABC pathway is highly significant in relation to the whole transcriptomic response of parasites exposed to delousing drugs. Interactome analysis showed that DM exposure promoted strong transcriptional signaling indicative of inhibitory components of the NOTCH/ABC pathway.

## 4. Materials and Methods

### 4.1. Experimental Design and High-Throughput Transcriptome Sequencing

From a recent study by our group, Illumina sequencing data for *C. rogercresseyi* challenged with DM and AZA were obtained from the Sequence Read Archive (Acc. No. SRX864101) [25]. Briefly, adult *C. rogercresseyi* were obtained from a commercial farm located in Puerto Montt, Chile. Individuals were transported on ice to the laboratory and maintained with a constant water flow at 12 °C and gentle aeration until the bioassays. The samples were separated into four glass containers: one control and three experimental. The bioassays were performed according to the procedures described by the SEARCH Consortium [26]. Transcriptome sequencing was conducted in unchallenged (control) *C. rogercresseyi* and in *C. rogercressey* challenged with DM (AlphaMax^®^ 1, 2, 3 ppb) and AZ (Bayer^®^ 1, 3, 10 ppb). Thirty sea lice adults (15 females and 15 males) were exposed to each concentration of DM and AZA in petri plates containing 50 mL of seawater. Each experiment was performed in triplicate. The exposure period to either DM or AZA was 40 and 30 min, respectively. During exposure, salmon lice were maintained at 12 °C. After 24 h, the organisms were fixed in RNAlater^®^ RNA Stabilization Reagent (Ambion^®^, Life Technologies™, Carlsbad, CA, USA) and stored at −80 °C until subsequent RNA extraction. The protocols for bioassays were performed according to the SEARCH Consortium [26]. Following DM (2 ppb) and AZA (3 ppb) exposure, twenty randomly selected individuals per concentration and drug were pooled. The drugs concentrations (DM, 2 ppb and AZA, 3 ppb) were selected by the transcriptomic performance shown in previous bioassays.

Total RNA was extracted from three pools of individuals by treatment using the RiboPure™ Kit (Ambion^®^, Life Technologies™) following the manufacturer’s instructions. Quantity, purity, and quality of isolated RNA were measured in the TapeStation 2200 (Agilent Technologies Inc., Santa Clara, CA, USA) using the R6K Reagent Kit according to the manufacturer’s instructions; samples with RIN over 8.0 were used for library preparation. Three biological replicates per pool (3 control, 3 DM, and 3 AZA) were used to construct the libraries. Subsequently, double-stranded cDNA libraries were constructed using the TruSeq RNA Sample Preparation Kit v2 (Illumina^®^). Libraries with mean length peaks above 290 ppb were used for sequencing and were quantified by qPCR using the Library Quantification Kit Illumina/Universal (Kappa, San Diego, CA, USA) according to the manufacturer’s instructions; each sample pool was sequenced using MiSeq (Illumina^®^, San Diego, CA, USA).

### 4.2. Identification of Putative NOTCH Signaling Pathway Transcripts and SNPs Mining

NOTCH signaling pathway transcripts were identified from the cDNA library of *C. rogercresseyi* [27]. The pathway components described for the mosquito *Anopheles gambiae*, deposited in the KEGG Pathway database, were used as a model to characterize the NOTCH signaling pathway of *C. rogercresseyi*. Contigs were annotated using the tBlastx algorithm from an enrichment crustacean EST-database (NCBI) with a cutoff *E*-value of 1 × 10^−5^. The identified structural motifs of NOTCH-like receptors were analyzed using the online ScanProsite tool. Using the assembly obtained from all identified NOTCH signaling pathway genes, SNPs mining was performed using the Genomics Workbench v8.1 software (CLC Bio., Aarhus, Denmark). The parameters used were as follows: window length = 11, maximum gap and mismatch count = 2, minimum average quality of surrounding bases = 15, minimum quality of central base = 20, maximum coverage = 100, minimum coverage = 8, minimum variant frequency (%) = 35, and maximum expected variations (ploidy) = 2.

### 4.3. RNA-seq Analysis Hierarchical Cluster Analysis

Sequencing data analysis was performed using the CLC Genomics Workbench software (CLC bio). Raw reads were filtered by quality and adapter/index trimmed. CLC bio’s *de novo* assembly algorithm was used to create a contig list from previously filtered reads using a mismatch cost = 2, insertion cost = 3, deletion cost = 3, length fraction = 0.8, similarity fraction = 0.8, and a minimum contig length = 250. Finally, 411 contigs were adjusted by mapped reads, and end gaps were treated as mismatches. Expression values were estimated as reads per kilobase of exon model per million mapped reads and then normalized to the total number of assembled contigs, using state numbers in reads per 1,000,000. This normalized the number of reads to the size of assembled contigs and allowed for assessing the transcripts that were upregulated among different groups. Hierarchical clustering was performed using the Euclidean distance matrix and complete linkage method, while the Manhattan metric distance was used for hierarchical cluster analysis. Analyses were conducted in the Cluster3/TreeView open software (3.0, Palo Alto, CA, USA).

### 4.4. Primer Design and Quantitative Real-Time Polymerase Chain Reaction Assay

Contig sequences annotated to *NOTCH, PSEN1, DELTEX, Aph -1, ABC* and *GROUCHO* for *C. rogercresseyi* were used as a template for primer design with the Primer3 Tool included in the Geneious Pro software version 8.0 [40] (the primer efficiencies are shown in Table S1). The qPCR runs were performed with StepOnePlus™ (Applied Biosystems^®^, Life Technologies) using the comparative ΔCt method. *β-tubulin* was selected as the housekeeping gene due to its stable value as inferred through the NormFinder algorithm. The other housekeeping genes assayed were *elongation factor alpha* and *beta actin*. Each reaction was conducted with a volume of 10 μL using the Maxima^®^ SYBR Green/ROX qPCR Master Mix (Thermo Scientific). The amplification cycle was as follows: 95 °C for 10 min, 40 cycles at 95 °C for 15 s, and 60 °C for 1 min, followed by a disassociation curve under these same conditions. The data obtained were analyzed through the Kruskal—Wallis test with Statistica software (Version 7.0, StatSoft, Inc.). Statistically significant differences were accepted with a *p* < 0.05.

### 4.5. Interactome, Principal Components, and Statistical Analyses

Complementary analysis was conducted with the ClusterMaker Cytoscape plugin [41] using the Markov Cluster algorithm to search protein-protein interaction network modules derived from tandem affinity purification/mass spectrometry. This approach clustered the network into modules based on purification enrichment score to indicate the strength of node association and provide a fixed set of genes with high protein-protein affinity (interactome cluster nodes). Principal component analysis using the Gene Ontology package showed two-dimensional views, retrieved by the DeDaL plugin of Cytoscape [42], that were used to visualize the relatedness of all RNA-seq samples. All *p*-values were adjusted with a false discovery rate correction for multiple testing by the Benjamini—Hochberg method [43]. All genes with false discovery rate-corrected *p*-values <0.05 were considered significant. The expression of genes found to be significantly different between both treatments were further characterized by a hierarchical clustering analysis. Hierarchical clustering was based on the expression pattern across the sampled population, thereby identifying clusters of genes with common expression profiles. Sample variances were homogeneous (normal distribution). The principal components were computed in DeDaL using singular value decomposition, a method allowing the use of missing data values without pre-imputation, as described in Gorban and Zinovyev [44]. Data points containing more than 20% of missing values were filtered from the analysis. DeDaL computes the first 10 principal components if there are more than 10 data points, and *k* principal components if there are k + 1 data points (*k* < 10). After computing the principal components, DeDaL reported the amount of variance explained by each of the principal components. DeDaL is the first method that permits constructing biological network layouts from high-throughput data using Cytoscape.

## 5. Conclusions

In conclusion, this study suggests that delousing drugs may increase the activity of inhibitory mRNA components, thereby promoting inhibitory NOTCH output signaling pathways. This study is the first to describe a correlated modulation of NOTCH signaling pathways and ABC transporter genes through the use of DM and AZA in *C. rogerresseyi*. Increasing chemicals concentrations were correlated with increased NOTCH mRNA expression, leading to interaction with ABC genes. Analyses emphasized that the key functions of NOTCH/ABC components were in regulating distinct phases of the delousing drug-response in *C. rogercresseyi*. It is likely that these components could be related to resistance modifications resulting from the frequent use of organophosphates and pyrethroids. In insects, the toxic effects of antiparasitics are well established, and the present results extend upon previous observations in sea lice species, in addition to suggesting that frequent delousing drug exposure promotes interactions with NOTCH/ABC genes (upregulation). However experimental studies are needed to elucidate the involvement of ABC transporters to pyrethroid or organophosphate resistance in *C. rogercresseyi*.

## Figures and Tables

**Table 1:** NOTCH pathway genes identified for *C. rogercresseyi* and differential transcription expressions between adults exposed to a delousing drug (azamethiphos (AZA) or deltamethrin (DM)) and the control group.

Name	Lowest *E*-Value	Accession (*E*-Value)	Description	Adults DM/Adults Control		Adults AZA/Adults Control	
				Fold Change	*p*-Value	Fold Change	*p*-Value
*ABCC V*	2 × 10^−33^	ETN58310.1	Multidrug resistance protein 2 (ATP-binding cassette protein c) [Anopheles darlingi]	0.83	0	0	0
*ABCC VI*	6 × 10^−98^	EHJ72514.1	ABC transporter family C protein ABCC2 [Danaus plexippus]	1.92	0	0.165	0
*ABCC VIII*	0	XP_004081991.1	PREDICTED: multidrug resistance-associated protein 4-like [Oryzias latipes]	−0.97	0	−1.6	0
*ABCC XI*	0	XP_008276233.1	PREDICTED: multidrug resistance-associated protein 4 [Stegastes partitus]	0.50	0	0.49	0
*ABCC IV*	3 × 10^−59^	EFX68457.1	ATP-binding cassette, sub-family C, member 4 [Daphnia pulex]	−0.58	0	NN	0
*ABCC IX*	0	KFQ99756.1	Multidrug resistance-associated protein 1 [Nipponia nippon]	1.18	0	0.7	0
*ABCC XIV*	3 × 10^−77^	XP_001862061.1	Multidrug resistance-associated protein 14 [Culex quinquefasciatus]	0.60	0	−0.4	0
*ABCB XII*	0	AHC54388.1	P-glycoprotein [Caligus rogercresseyi]	−0.58	0	−0.61	0
*ABCC III*	1 × 10^−58^	XP_001022386.1	ABC transporter family protein [Tetrahymena thermophila]	NN	0	0	1
*ABCB VI*	1 × 10^−39^	WP_018508877.1	ABC transporter [Thiobacillus thioparus]	0	1	0	1
*ABCC I*	1 × 10^−40^	EAS00380.2	ABC transporter C family protein [Tetrahymena thermophila SB210]	0	1	0	1
*ABCB VII*	1 × 10^−45^	WP_016832902.1	Amino acid ABC transporter ATP-binding protein [Herbaspirillum lusitanum]	0	1	0	1
*ABCC II*	3 × 10^−100^	WP_014005418.1	Multidrug ABC transporter ATP-binding protein [Collimonas fungivorans]	0	1	0	1
*ABCB VIII*	1 × 10^−6^	WP_005919053.1	Excinuclease ABC subunit A, uvrA [Pediococcus acidilactici]	0	1	0	1
*ABCB IX*	2 × 10^−43^	WP_008117112.1	ABC transporter [Herbaspirillum sp. YR522]	0	1	0	1
*ABCB I*	1 × 10^−112^	WP_014007698.1	Methionine ABC transporter ATP-binding protein [Collimonas fungivorans]	0	1	0	1
*ABCB II*	4 × 10^−78^	WP_027616274.1	ABC transporter ATP-binding protein [Pseudomonas sp. URHB0015]	0	1	0	1
*ABCB III*	4 × 10^−128^	WP_007878001.1	Peptide ABC transporter ATP-binding protein [Herbaspirillum sp. CF444]	0	1	0	1
*ABCB XI*	0	EKC24099.1	ATP-binding cassette sub-family B member 8, mitochondrial [Crassostrea gigas]	0.07	4.7 × 10^−11^	−0.41	0
*ABCB IV*	1 × 10^−53^	CCV01190.1	ABCB/P-glycoprotein-like protein [Mytilus galloprovincialis]	0	1	0	1
*ABCC XV*	0	XP_005496337.1	PREDICTED: multidrug resistance-associated protein 4-like [Zonotrichia albicollis]	0.78	0	−0.43	0
*ABCB V*	2 × 10^−74^	KFM56907.1	Multidrug resistance protein 1 [Stegodyphus mimosarum]	0	1	0	1
*ABCC XII*	0	KDR17053.1	Multidrug resistance-associated protein 7 [Zootermopsis nevadensis]	0.28	0	0.28	0
*ABCC XIII*	0	XP_004342702.1	Multidrug resistance-associated protein 3 [Capsaspora owczarzaki ATCC 30864]	0.42	0	−0.144	0
*ABCB X*	2 × 10^−24^	EFN74443.1	ATP-binding cassette sub-family B member 10, mitochondrial [Camponotus floridanus]	NN	0	0	1
*ABCC X*	0	EFX68457.1	ATP-binding cassette, sub-family C, member 4 [Daphnia pulex]	2.08	0	0.151	0
*ABCC VII*	2 × 10^−172^	XP_002116818.1	ATP-dependent bile acid permease [Aedes aegypti]	1.76	0	0.116	0
*DELTEX*	8 × 10^−60^	EGI67423.1	Protein deltex [Acromyrmex echinatior]	0.135	0	−0.007	6.95 × 10^−10^
*FRINGE*	7 × 10^−96^	XP_002422797.1	Fringe glycosyltransferase, putative [Pediculus humanus corporis]	0.130	8.88 × 10^−11^	−0.531	0
*Aph-1*	3 × 10^−111^	ADD24087.1	Gamma-secretase subunit Aph-1 [Lepeophtheirus salmonis]	0.43	2.62 × 10^−05^	−0.55	0
*GROUCHO1*	0	BAN21009.1	Groucho [Riptortus pedestris]	0.01	0.48	0.541	0
*GROUCHO2*	0	BAN21009.1	Groucho [Riptortus pedestris]	−0.29	0	0.57	0
*HDAC*	2 × 10^−29^	XP_001862395.1	Histone deacetylase [Culex quinquefasciatus]	−0.167	0	0.58	0
*HDAC3*	1 × 10^−158^	EFX81904.1	Putative histone deacetylase HDAC3 protein [Daphnia pulex]	0.232	0	0.25	0
*HDAC4*	3 × 10^−148^	XP_001605910.2	PREDICTED: histone deacetylase 4-like [Nasonia vitripennis]	−0.752	0	0.39	0
*HDAC6*	0	NP_001162760.1	HDAC6, isoform D [Drosophila melanogaster]	−0.51	0	−1.0 × 10^−1^	0
*NOTCH1*	0	EFX77274.1	NOTCH 2 [Daphnia pulex]	0.609	0	0.114	0
*NOTCH2*	0	AGT57827.1	NOTCH [Euperipatoides rowelli]	0.42	0	0.104	0
*NOTCH3*	1 × 10^−36^	EZA58452.1	Neurogenic locus NOTCH protein [Cerapachys biroi]	−0.181	0	−0.51	0
*NUMB*	1 × 10^−106^	EZA52633.1	Protein numb [Cerapachys biroi]	−0.75	0	0.03	0
*PSEN*	2 × 10^−134^	EHJ71857.1	Presenilin-like signal peptide peptidase [Danaus plexippus]	0.391	0	−0.085	0
*SKIP*	1 × 10^−31^	AGM16247.1	Even-skipped isoform 2 [Nasonia vitripennis]	NN	0	0	1

**Table 2:** (**A**) Survival rate of sea lice individuals exposed to deltamethrin and (**B**) azamethiphos.

(A) Deltamethrin (ppb)	Female	Male
0	100	100
1	53.3	13.3
2	26.7	20.0
3	26.7	0.0
**(B) Azamethiphos (ppb)**	**Female**	**Male**
0	100	100
1	80	73.3
3	53.3	40
10	12.4	10
30	0	0

**Table 3:** List of single nucleotide polymorphisms (SNPs) identified in NOTCH pathway genes in adult *C. rogercresseyi*.

Name	Consensus Position	Allele	Frequency	Substitution
*DELTEX*	3468	A/G	31.30	3′UTR
	3079	G/A	30.94	3′UTR
	3067	G/T	26.21	3′UTR
	2784	G/T	31.03	Synonymous
	2698	C/T	20.88	Synonymous
	2679	T/C	32.35	Synonymous
	2616	T/C	43.70	Synonymous
	2434	A/G	46.15	Non synonymous
	2181	T/C	47.35	Synonymous
	1853	C/A	21.05	Non synonymous
	1659	A/C	41.38	Non synonymous
	1301	T/C	31.39	Synonymous
	1116	A/G	34.81	Synonymous
	1038	C/T	27.01	Synonymous
	987	G/A	48.63	Synonymous
	954	G/A	34.18	Synonymous
	927	A/G	38.46	Synonymous
	489	A/G	25.50	5′UTR
*FRINGE*	133	T/A	54.55	5′UTR
	940	C/T	29.27	Non synonymous
	979	A/C	49.04	Non synonymous
	1260	T/C	45.45	5′UTR
*APH-1*	829	T/C	42.86	Synonymous
	793	T/G	48.06	Synonymous
	671	T/C	21.16	Synonymous
*GROUCHO1*	1754	C/T	25.73	Synonymous
	1430	T/C	23.83	Synonymous
	1202	G/A	20.94	Synonymous
	266	G/A	35.27	Synonymous
*HDAC6*	3792	T/C	45.71	3′UTR
	3433	C/T	41.89	3′UTR
	3381	C/G	34.60	3′UTR
	3156	G/T	38.60	3′UTR
	2586	T/C	39.13	Synonymous
	2199	T/C	33.71	Synonymous
	681	A/C	21.52	Synonymous
	600	T/C	24.69	Synonymous
*NOTCH1*	10732	C/A	48.88	Synonymous
	3509	G/A	24.35	Non synonymous
	3067	C/T	26.40	Synonymous
	2890	A/G	20.35	Synonymous
	2758	C/T	41.67	Synonymous
	2491	G/C	28.57	Synonymous
	2035	A/C	30.59	Synonymous
	1468	G/T	36.89	Synonymous
	400	C/A	44.83	Synonymous
	281	T/A	25.93	5′UTR
	251	C/T	31.37	5′UTR
*NOTCH2*	373	A/C	21.32	Non synonymous
*PSEN*	1463	T/C	48.50	3′UTR
	797	T/C	21.07	Non synonymous
	352	T/G	24.98	Non synonymous
*NUMB*	51	G/A	27.27	Synonymous

## Supplementary Materials

Supplementary materials can be found at http://www.mdpi.com/1422-0067/17/5/304/s1.

## Abbreviations

ABC	ATP-binding cassettes
AZA	azamethiphos
CSL	CBF1/RBP-Jk/Suppressor of Hairless/LAG-1
DM	deltamethrin
HDAC	histone deacetylase
N^IC^	intracellular C-terminal fragment of NOTCH
PCA	principal components analysis
PSEN1	presenilin-1
SNPs	single nucleotide polymorphisms
