# Supplementary material for: Evidence for the Induction of Key Components of the NOTCH Signaling Pathway via Deltamethrin and Azamethiphos Treatment in the Sea Louse Caligus rogercresseyi
Source: Int J Mol Sci. 2016 May 12;17(5):304. doi: 10.3390/ijms17050304 (PMC4881433; doi:10.3390/ijms17050304)
Supplement: Supplementary file 1 [file ijms-17-00304-s001.pdf]

Supplementary Materials: Evidence for the Induction of Key Components of the Notch Signaling Pathway via Deltamethrin and Azamethiphos Treatment in the Sea Louse *Caligus rogercresseyi*

Sebastian Boltaña, Jaqueline Chávez-Mardones, Valentina Valenzuela-Muñoz and Cristian Gallardo-Escárate

Table S1. Primers designed for Notch signaling pathways from *C. rogercresseyi*.

Gene Primers	Sequence (5′–3′)	Efficiency (%)
GROUCHO_F	CCGATCCACGCCCCAGCAGCAGA	86.7
GROUCHO_R	GGGGGAGGGATCCGTTGGGCGTG	
Aph – 1_F	AGCGGGACGACGGCAAACCACCA	89.2
Aph – 1_R	TTGACGGCCCTTGGGGCTCCCTC	
NOTCH i_F	TGGCACTACAAGAGAGGCGGAGC	87.9
NOTCH i_R	TGACGACTTCTACTGTCGCTGCCC	
PSEN_F	ACGGAGCGAAAGGAGCCCACGAA	86.7
PSEN_R	AACGCAACGGCTGGCTCTCCGGT	
DELTEX_F	TGAGGAGGCTGAGCGGCGAAGGA	87.3
DELTEX_R	TCCACAAACCTATGGACAGCAGCAGCC	
ABCB XII_F	GAGTCCCGGCGTGCGTCCTCAGT	97.1
ABCB XII_R	GGCCTCTGTGGTCATGGGGGCCT	
ABCC XII_F	CCGCTGAAAATGAAGGGCTCCTGGGGT	93.2
ABCC XII_R	TCGCGGCCCTCTTTCGATTGTCGAGC	
ABCC XIII_F	TGTCAGTGGATGTCTCTCGGATCGCA	89.5
ABCC XIII_R	TGCCTCCAATGATGGCCCAGCCCAG	
